# Impact of artificial aging on the color stability and translucency of ceramic laminate veneers using different glass and hybrid ceramic materials

**DOI:** 10.2340/biid.v13.45798

**Published:** 2026-04-23

**Authors:** Ahmed Shams, Nesma Elgohary

**Affiliations:** Fixed Prosthodontics Department, Faculty of Dentistry, Mansoura University, Mansoura, Egypt

**Keywords:** ceramic veneers, artificial aging, color stability, translucency, IPS e, max CAD, Cerec Tessera, Cerasmart, Vita Enamic

## Abstract

**Background:**

The color stability and translucency of contemporary Computer-Aided Design/Computer-Aided Manufacturing (CAD/CAM) glass and hybrid ceramic materials used for minimally invasive laminate veneers remain a clinical concern, particularly after exposure to aging conditions simulating oral service. The purpose of this *in vitro* study was to investigate the influence of accelerated artificial aging on the color stability and translucency of ceramic laminate veneers fabricated from different glass and hybrid ceramic materials.

**Materials and methods:**

A total of 40 composite resin discs (A2 dentin shade, 8.0 mm diameter × 4.0 mm thickness) were fabricated to simulate the normal dentin substrate. They were randomly assigned to four groups (*n* = 10) according to the ceramic veneering material: (EC) IPS e.max CAD, (CT) Cerec Tessera, (VE) Vita Enamic, and (CS) Cerasmart. Then, 40 disc-shaped ceramic veneers (0.5 mm thickness) were fabricated and adhesively cemented to the substrates. Baseline color and translucency parameters were measured using a digital spectrophotometer. After artificial thermomechanical aging, color differences (ΔE00), using the CIEDE2000 formula and translucency parameter (TP) were calculated. The resulting data were statistically analysed using repeated-measures two-way analysis of variance (ANOVA) test (material × aging), followed by Post hoc Tukey test for multiple-group comparisons and Paired *t*-test for within-group comparisons, at *p*-value ≤ 0.05.

**Results:**

Artificial aging significantly affected color stability and translucency for all tested materials (*p* < 0.001). Glass ceramics (EC and CT) demonstrated lower ΔE₀₀ values within clinically acceptable limits, whereas hybrid ceramics (VE and CS), particularly CS, exhibited significantly higher color changes exceeding acceptability threshold. Translucency significantly decreased after aging for all materials (*p* < 0.001).

**Conclusions:**

Artificial thermomechanical aging adversely affected the optical properties of all tested ceramic laminate veneers. The hybrid ceramic materials, particularly CS, were the most affected by aging in terms of color stability, whereas the glass ceramic materials (EC and CT) exhibited superior color stability. A reduction in translucency was observed for all materials after aging.

**KEY MESSAGES**

1. Thermomechanical aging impacts esthetics: Accelerated artificial aging significantly alters both color stability and translucency of ceramic laminate veneers.
2. Glass ceramics outperform hybrid ceramics: IPS e.max CAD and Cerec Tessera maintain superior color stability over hybrid ceramics, with color changes remaining clinically acceptable.
3. Hybrid ceramics are more vulnerable: Materials like Cerasmart show greater susceptibility to aging-induced color shifts and loss of translucency.
4. Translucency declines across materials: All tested materials exhibit a significant reduction in translucency after artificial aging , emphasising the importance of material selection for long-term esthetic outcomes.

## Introduction

Restorative esthetic dentistry should be performed as conservatively as possible. Given modern advancements, clinicians should select techniques and materials that provide the most conservative restorative approach. Furthermore, they should ascertain the biological and mechanical requirements that fulfil clinical durability while satisfying the esthetic desires of patients [1]. Laminate veneers are considered the most popular treatment option for unesthetic tooth restoration. The challenge in ceramic veneers lies in attaining optimal esthetics by harmonising shade matching with the provision of a thin ceramic restoration. The ceramic veneer restoration color is influenced by various factors, including restoration thickness, cement color, and tooth color [2].

Over the last 30 years, CAD/CAM techniques have been promoted, and ceramic laminate veneers have been subjected to both a material and a technological revolution [1]. CAD/CAM restoration quality is exceptionally high due to the precision of measurements and manufacturing processes. The milled ceramic blocks exhibit a natural appearance because of their enamel-like translucent properties, and they are available in a diverse array of shades [3].

Lithium disilicate ceramics are developed for CAD/CAM dental prostheses [4]. They were initially designed for use with press technology (IPS Empress 2) and later succeeded by enhanced versions, including IPS e.max CAD and IPS e.max Press. IPS e.max CAD is supplied in a metasilicate condition, consisting of 40% lithium metasilicate crystals with platelet shapes dispersed in a bluish glassy matrix. To obtain the final tooth shade and lithium disilicate structure, a crystallisation process is needed, which includes a firing cycle at 840°C for 25 minutes [5]. Earlier research reported that the lithium disilicate ceramics’ flexural strength ranges from 300 to 520 MPa, with the survival rate of restoration varying between 96 and 100% over a 3-year period [4].

An innovative advanced lithium disilicate (ALD) ceramic was developed in 2021 by Dentsply Sirona called Cerec Tessera. It is a high-strength, tooth-colored glass-ceramic block that necessitates obligatory firing to attain its final strength. It is characterised by a unique microstructure. The manufacturer states that the material is composed of lithium disilicate and virgilite, which is lithium aluminium silicate dispersed within a glassy matrix enhanced with zirconia. Additional crystals of virgilite grow during the firing process. The rod-shaped crystals of lithium disilicate improve tensile strength, thus preventing crack propagation. The tiny crystals of virgilite that formed during the firing process increase pre-compression stress. So, they play an important role in achieving a biaxial flexural strength that is greater than 700 MPa [6].

Until now, research concerning the longevity of hybrid ceramic restorations is still limited. Hybrid ceramics are composed of resin nano-ceramic (RNC) materials and polymer-infiltrated ceramic network (PICN) [7]. The PICN ceramic material is employed, utilising CAD/CAM technology. It is composed of polymer organic material within a ceramic inorganic matrix [8]. Vita Enamic is considered the material of choice of the hybrid ceramic materials (86 wt.% dominant ceramic mesh reinforced by 14 wt.% polymer), with specific mechanical and optical properties. Owing to the polymeric matrix, it provides 30 GPa modulus of elasticity which is extremely close to dentin (15–20 GPa), and 150–160 MPa flexural force resistance. It can be used for minimally invasive restorations such as, overlays, onlays, inlays, veneers, and crowns, with implants and natural teeth [7, 8].

RNC materials are hybrid materials having optical and physical characteristics that merge the properties of dental ceramics with properties of polymer materials. They represent a polymeric organic matrix infiltrated with inorganic ceramic crystals, such as zirconium dioxide and quartz [7]. Shofu Block HC (Shofu), Cerasmart (GC), Lava Ultimate (3M ESPE), Katana Avencia Block (Kuraray Noritake), and Grandio Blocs (VOCO) are examples of this category. Cerasmart is composed of barium glass and silica filler particles dispersed in an organic resin matrix with up to 71% by its weight and has been found to have a flexural strength of 230 MPa [9].

The esthetic success of restorations relies mainly upon the stability of color of the used dental restorations [10]. Accelerated artificial aging (AAA) is a technique that simulates clinical scenarios, enabling the assessment of color change of materials over time. This technique was released to examine the stability of color for various dental materials, including resin cements [11].

Achieving excellent esthetic restorations requires a thorough understanding of fundamental color perception, as well as the optical and structural properties of both dental materials and teeth [12]. The Commission Internationale de l’Eclairage (CIE, International Commission on Illumination) suggested various color systems, with the most commonly utilised, being the Commission Internationale de l’Éclairage (CIE) L* a* b* color space (CIELAB). It was launched in 1976 to offer consistent and uniformly perceived color differences. Within it are 3 axes which are L*, a*, and b*, representing the numerical description of color [13]. Within dental research, different color difference formulas are designed to provide a quantitative representation of the perceived color difference between two objects [14]. Although the CIELAB color-difference formula (ΔE*) has been widely used in the dental field, several advanced color difference formulas including CIEDE2000 (ΔE_00_) have been developed to make a single number shade pass/fail equation for evaluation of color differences [15]. The CIEDE2000 formula has a better correlation with human color perception compared to the CIELAB formula [16]. The translucency parameter (TP) is utilised to evaluate the restorations’ translucency. It is calculated from the color difference of a certain specimen against black and white backgrounds [13].

Although it is well established that glass ceramics exhibit superior chemical and color stability compared with resin-based and hybrid materials, the color stability of ceramic materials used for minimally invasive laminate veneers with different chemical compositions remains uncertain. Moreover, the longevity of restoration color may be altered after clinical service, and limited data are available regarding the optical behaviour of novel ceramics, such as Cerec Tessera, after thermomechanical agiing. Therefore, further investigation is required to clarify the long-term optical performance of both hybrid and newly developed glass ceramic materials [12].

This *in vitro* study was performed to investigate the effect of AAA on the color stability and translucency of minimal-thickness ceramic laminate veneers using different glass and hybrid ceramic materials. The null hypothesis stated that artificial aging would not significantly impact the stability of color and translucency of all tested ceramic laminate veneer materials.

## Materials and methods

### Ethical approval

This *in vitro* study protocol followed all guidelines provided by the Local Research Ethics Committee of the Faculty of Dentistry, Mansoura University, and received approval no. R.25.05.6.

### Sample size calculation

Based on data reported by Chen et al. [2], a power analysis test was performed for calculating sample size using the G* power program version 3.1.9.7. Based on an effect size of 0.42 using a 2-tailed test, an α error of 0.05 and a power of 80.0%, the total calculated sample size of 40 was divided into four main groups (*n* = 10).

### Fabrication of composite resin background discs

To replicate the natural background of dentin, 40 composite resin discs of A2 dentin shade (CLEARFIL PLUS, Kuraray, Japan) were constructed using a Teflon custom-made circular mould with the following dimensions: 8.0 mm diameter × 4.0 mm thickness [2]. The discs were light-cured using an Light Emitting Diode (LED) light-curing unit (Elipar DeepCure-S, 3M ESPE Dental, MN, USA) with an irradiance of 1400 mW/cm^2^ for a time of 40 seconds on either side of each disc, according to the manufacturer’s instructions. For obtaining a uniform bonding surface, silicon carbide wet paper (800-grit) was utilised, and the discs’ thickness was adjusted to 4.0 ± 0.02 mm. The thickness of the composite resin background discs was assessed through five-point measurements by the aid of a digital stainless steel caliper (100 mm/4 in Digital Gauge Vernier Caliper, Hanhe Co. Ltd., China).

### Specimens grouping

According to the ceramic material used for laminate veneer fabrication, the 40 composite background discs were allocated randomly into four equal groups (*n* = 10): group EC: 10 veneers from lithium disilicate glass ceramic material (IPS e.max CAD, Ivoclar Vivadent, Schaan, Liechtenstein), group CT: 10 veneers from ALD glass ceramic material (Cerec Tessera, Dentsply Sirona, NC, USA), group VE: 10 veneers from PICN hybrid ceramic material (Vita Enamic, Vita Zahnfabrik, Bad Säckingen, Germany), and group CS: 10 veneers from RNC hybrid ceramic material (Cerasmart, GC Corp, Tokyo, Japan). All CAD/CAM ceramic veneer materials utilised in this study are listed in Table 1.

**Table 1:** CAD/CAM ceramic veneer materials used in this study.

Group	*n*	Material	Product name	Composition	Manufacturer
**EC**	10	Lithium disilicate glass ceramic	IPS e.max CAD (HT A2/C14)	SiO_2_ (57–80%) K_2_O (< 13%) Li_2_O (11–19%) MgO (< 5%) ZrO_2_ (< 8%) P_2_O_5_ (< 11%) ZnO (< 8%) Al_2_O_3_ (< 5%) coloring oxides (< 0.8%)	Ivoclar Vivadent, Schaan, Liechtenstein
**CT**	10	Advanced lithium disilicate ceramic	Cerec Tessera (HT A2/C14)	SiO_2_ (57–80%) K_2_O (0–13%) Li_2_O (11–19%) P_2_O_5_ (0–11%) ZnO (0–8%) ZrO_2_ (0–8%) other oxides (0–12%)	Dentsply Sirona, NC, USA
**VE**	10	Polymer-infiltrated ceramic-network	Vita Enamic (2M2-HT/EM-14)	- Inorganic component (86 wt%): (58–63%) SiO_2_ (9–11%) Na_2_O (20–23%) Al_2_O_3_ (4–6%) K_2_O (< 1%) ZrO_2_ (0.5–2%) B_2_O_3_ (< 1%) CaO - Organic component (14 wt%): TEGDMA, UDMA	Vita Zahnfabrik, Bad Säckingen, Germany
**CS**	10	Resin nano- ceramic	Cerasmart 270 (A2 HT/14)	- Inorganic component (71 wt%): SiO_2_, barium glass - Organic component (29 wt%): UDMA, Bis-MEPP, polyfunctional methacrylate	GC Corp, Tokyo, Japan

UDMA: urethane dimethacrylate; TEGDMA: triethylene glycol dimethacrylate; Bis-MEPP: bisphenol A ethoxylate dimethacrylate; EC: IPS e.max CAD; CT: Cerec Tessera; VE: Vita Enamic; CS: Cerasmart.

### Fabrication of ceramic veneers

Based on specimens grouping, the tested ceramic veneers were fabricated through CAD/CAM technique, following the manufacturer’s recommendations. A total of 40 disc-shaped ceramic specimens (0.5 mm thickness × 8.0 mm diameter) were made. First, a standardised cylindrical design with 8.0 mm diameter was performed for all tested materials using a CAD software (DentalDB 3.1 Rijeka, exocad GmbH, Darmstadt, Germany). After adjusting the design virtually, it was nested by the aid of a dental CAM system (iCAM V5 Smart, imes-icore GmbH, Eiterfeld, Germany). Once the tool path calculation was completed, it was transferred into the milling machine through the order button ‘mill’ integrated in the software programme, and then the milling process started. A five-axis dry/wet milling machine (CORiTEC 250i touch, imes-icore GmbH, Germany) was used with the suitable milling tools for both gross milling and fine adjustment.

The finished cylindrical designs for both glass ceramic groups EC and CT were wet-milled from the corresponding block material: IPS e.max CAD (HT A2/C14) for group EC and Cerec Tessera (HT A2/C14) for group CT. The finished designs for both hybrid ceramic groups VE and CS were dry-milled using milling blocks with appropriate thickness: Vita Enamic (2M2-HT/EM-14) for group VE and Cerasmart 270 (A2 HT/14) for group CS. After completing milling, a linear precision diamond saw blade (15LC, BUEHLER, USA) fitted into a water-cooled, low-speed (2500 rpm with 6 mm/min feed rate) sectioning machine (IsoMet 4000, BUEHLER, USA) was used for sectioning/slicing each milled cylinder into multiple disc-shaped ceramic specimens with uniform dimensions of 8.00 mm diameter and 0.5 mm thickness.

The post-sectioning crystallisation and glazing procedures were performed for the glass ceramic disc specimens, following the manufacturer’s guidelines using a compatible ceramic furnace with controlled long-term cooling and vacuum functions (Programat P500, Ivoclar Vivadent, Schaan, Liechtenstein). For sectioned hybrid ceramic discs, only finishing and high-gloss polishing were completed using the manufacturer-recommended finishing/polishing tools while low-speed and light pressure were applied. For Vita Enamic veneers; special instruments of a two-step Vita Enamic polishing system (VITA ENAMIC Polishing Sample Set, #232097, Vita Zahnfabrik, Bad Säckingen, Germany) were used for finishing and successful high-gloss polishing. Firstly, according to the manufacturer’s recommendations, pre polishing with the pink polishers of the polishing system while cooling with water was done. Then, high-gloss polishing with the grey diamond coated polishers at a lower speed and without water cooling was performed. For Cerasmart 270 veneers, initial finishing was performed using medium-grit silicone polishers (Ultimate F&P Kit, GC Corp, Tokyo, Japan), followed by fine-grit silicone polishers from the same system to further refine the surface. For high-gloss polishing, a special polishing paste (DiaPolisher Paste, GC Corp, Tokyo, Japan) was applied using a soft GC goat-hair brush at low speed until a smooth, glossy surface was obtained. All finished/glazed discs were checked using the digital caliper to check and confirm the uniform dimensions and then cleaned by gentle steam blasting and finally dried.

### Cementation of ceramic veneers to composite background discs

For cementation procedure, a 37% phosphoric acid gel (N-Etch Etching Gel, Ivoclar Vivadent, Schaan, Liechtenstein) was used to etch surfaces of composite background discs for 15 seconds, followed by rinsing with water for 30 seconds and gentle drying with air for 15 seconds. Then, a thin layer of light-cured universal adhesive (All-Bond Universal, Bisco, IL, USA) was utilised, air-thinned, and light-cured for 10 seconds utilising an LED light-curing unit (Elipar DeepCure-S, 3M ESPE Dental, MN, USA) with irradiance of 1400 mW/cm^2^ [17].

Regarding the ceramic veneers, their surfaces were treated following the manufacturers’ recommendations. For IPS e.max CAD and Cerec Tessera veneers, the dried surfaces were treated with 9.5% hydrofluoric acid etching gel (Porcelain Etchant, Bisco, IL, USA) for 20 seconds for IPS e.max CAD and 30 seconds for Cerec Tessera, while 5.0% hydrofluoric acid gel (IPS Ceramic Etching Gel, Ivoclar Vivadent, Schaan, Liechtenstein) was used for treatment of Vita Enamic and Cerasmart veneers, using disposable brushes, for 60 seconds. All veneers were thoroughly rinsed under running water for 60 seconds and subsequently dried by air for 20 seconds. Then, all etched veneers were silanised using a single-component silane coupling agent (Porcelain Primer, Bisco, IL, USA) applied with a brush for 30 seconds and blown carefully by a light stream of air for 5 seconds.

A light-cured, x-ray opaque, syringeable composite resin luting cement (VITIQUE esthetic resin cement, Transparent, DMG Chemisch-Pharmazeutische Fabrik GmbH, Hamburg, Germany) was used for the cementation procedure. A proper amount of cement was applied and distributed evenly on the ceramic veneer disc bonding surface [2]. Each ceramic veneer disc with its cement was held using a plastic adhesive pick up stick (OptraStick, Ivoclar Vivadent, Schaan, Liechtenstein). Then, it was carefully positioned on its respective composite disc with minimal finger pressure to ensure proper seating. A disposable brush was used to remove excess cement. After that, each paired specimen was fitted into a specially fabricated loading apparatus to ensure standardised load application during the cementation process, thereby ensuring a consistent thickness of the cement layer. Using the same LED unit, following the manufacturer’s guidelines, initial light-curing was performed for 2 seconds to allow easy removal of excess cement using a scaler following the cement’s initial setting. The light-curing step was completed for 40 seconds, followed by finishing and polishing of cementation lines. Finally, all paired specimens were preserved in a solution of saline at room temperature for 1 day to ensure full setting of the luting cement prior to testing [2, 17].

### Color measurements after ceramic veneers’ cementation

Color measurements were obtained using a calibrated digital spectrophotometer (VITA Easyshade V, VITA Zahnfabrik, Germany). All measurements were performed by a single, highly experienced operator, possessing nearly 10 years of expertise in dental materials research and extensive training with the Easyshade device, under standardised conditions in accordance with the manufacturer’s instructions. The specimens of each group were firstly numbered from 1 to 10 and then measured consecutively at a consistent time of day to minimise variability due to time-dependent factors such as light conditions, temperature, or operator performance. The spectrophotometer measuring probe tip was applied perpendicular to the centre of the specimen. For each specimen, three consecutive measurements were recorded and averaged to ensure repeatability and reliability. The spectrophotometer was calibrated every three measurements, following the manufacturer’s recommendation. Measurements of color parameters were performed against standardised backgrounds with known CIE Lab* values. A neutral grey background (L* = 50.0, a* = 0.0, b* = 0.0) was used for color measurements, while standardised white (L* = 96.0, a* = 0.0, b* = 1.0) and black (L* = 4.0, a* = 0.0, b* = 0.0) backgrounds were used for TP calculation. These values are consistent with those reported in previous dental color studies and fulfil the requirements for valid color and TP assessment [16, 18, 19]. The resulted baseline (non-aged) measurements served as the control condition.

The TP was obtained by estimating the color difference in each specimen against black and white backgrounds with the following formula:

TP = {(LB*−LW*)^2^ + (aB*−aW*)^2^ + (bB*−bW*)^2^}^1/2^

where TP is the translucency parameter (0–100); the higher the TP value, the more translucent the object, L* value indicates lightness, while a* value indicates the red-green axis and b* value indicates the yellow-blue axis, B is for the color parameter against a black background, and W is for the color parameter against a white background [2, 17, 20].

### Fatigue aging

All specimens were subjected to a combined thermomechanical aging protocol, consisting of thermal cycling followed by mechanical loading.

#### Thermal cycling testing (thermal aging)

To mimic the thermal alternations and stresses that happen in the oral environment, all specimens underwent an AAA process using a thermal cycling apparatus (thermocycler, SD Mechatronik, Feldkirchen-Westerham, Germany). In this study, thermal cycling was performed for 10,000 cycles alternating between 5 and 55°C with 30 seconds dwell time in each distilled water bath and a transfer time of 5 seconds. According to the latest International Standardization Organization specifications (ISO/TS11405:2015), this aging protocol is comparable to roughly 1 year of use in the oral cavity [21, 22].

#### Dynamic loading testing (mechanical aging)

All specimens were subjected to artificial mechanical cycling fatigue, simulating 1 year of clinical service using a computer-controlled chewing simulator machine (Chewing Simulator CS-4.4, SD Mechatronik, Germany). Specimens were submitted to 240,000 load cycles with 1.6 Hz frequency to replicate an intermittent unidirectional axial load of 50 Newton. Load was applied with a steatite-ceramic, enamel-like antagonist. The integrity of air-dried specimens was monitored at regular intervals visually under good illumination and tactilely with a dental probe to detect any mode/sign of early failure (chipping, crack, debonding, or fracture) [23, 24].

### Color measurements after fatigue aging

After completion of all fatigue cycles, a second color measurement was conducted for all specimens. Utilising the same protocol and under the same conditions as previously followed for the initial measurements, the color parameters for all specimens against neutral grey, white, and black backgrounds were obtained and recorded. The TP was also estimated after fatigue aging. Color differences (ΔΕ values) for all specimens between before (baseline, control) and after fatigue aging were calculated according to the CIEDE2000 formula using the following color difference (ΔE_00_) equation:

ΔE_00_ = [(ΔL/K_L_ S_L_)^2^ + (ΔC/K_C_ S_C_)^2^ + (ΔH/K_H_ S_H_)^2^ + RT (ΔC/K_C_ S_C_) (ΔH/K_H_ S_H_)]^1/2^

where ΔL, ΔC, and ΔH are the differences in lightness, chroma, and hue, and RT (rotation function) is a function that accounts for the interaction between chroma and hue differences in the blue region. S_L_, S_C_, and S_H_ are the weighting functions for the lightness, chroma, and hue components, respectively. K_L_, K_C_ and K_H_ are the parametric factors according to different viewing parameters that were set to 1. The ΔE_00_ values were assessed based on clinical perceptibility and acceptability thresholds set at ΔE_00_ = 1 and 3.3, respectively [16, 25].

### Statistical analysis

Data were organised, coded, and statistically analysed using IBM SPSS statistics software (version 22; IBM Corp., New York, USA). Quantitative data were presented as mean values ± standard deviations after confirming normal distribution using the Shapiro-Wilk test. Repeated-measures two-way analysis of variance (ANOVA) test (material × aging) was performed to evaluate the effects of material type, aging, and their interaction on color difference and TP, followed by Post hoc Tukey test for multiple-group comparisons. Within-group comparisons between baseline and post-aging measurements were performed using Paired *t*-test. A *p*-value ≤ 0.05 was considered statistically significant.

## Results

### Influence of material-aging interaction on color stability and translucency

Repeated-measures two-way ANOVA test was performed to evaluate the effects of material type (between-groups factor), aging condition (within-group factor), and their interaction (material × aging) on ΔE₀₀ and TP values. The analysis revealed a statistically significant main effect of material type on ΔE₀₀ values (*p* < 0.001), indicating that color stability significantly differed among the tested ceramic materials. A statistically significant effect of aging was also observed (*p* < 0.001), confirming that artificial aging significantly influenced color change regardless of material type. In addition, a statistically significant interaction between material type and aging condition was detected for ΔE₀₀ (*p* < 0.001). This interaction indicates that the magnitude of color change induced by aging was material-dependent, with hybrid ceramic materials exhibiting greater susceptibility to aging-related discoloration compared with glass ceramic materials (Table 2).

**Table 2:** Results of repeated-measures two-way ANOVA test evaluating the effects of material type, aging , and their interaction on color difference (ΔE₀₀) and translucency parameter.

Parameter	Source	Type III sum of squares	df	Mean square	*F*	*P*
**ΔE₀₀**	Material	20.40	3	6.80	495.78	*p* < 0.001*
	Aging	0.67	1	0.67	16.49	*p* < 0.001*
	Material × Aging	0.98	3	0.33	23.83	*p* < 0.001*
**TP**	Material	5.48	3	1.83	196.27	*p* < 0.001*
	Aging	0.21	1	0.21	7.67	*p* < 0.001*
	Material × Aging	0.35	3	0.12	12.68	*p* < 0.001*

* Significance at *p*-value ≤ 0.05. ANOVA: analysis of variance; TP: translucency parameter

Similarly, repeated-measures two-way ANOVA test demonstrated a statistically significant main effect of material type on TP values (*p* < 0.001), reflecting inherent differences in translucency among the tested materials. Aging also exerted a statistically significant effect on TP (*p* < 0.001), leading to a reduction in translucency for all materials after thermomechanical aging. Furthermore, a statistically significant interaction effect between material type and aging was observed for TP (*p* < 0.001), indicating that the degree of translucency reduction varied according to the ceramic material. These findings confirm that both color stability and translucency of ceramic laminate veneers were influenced not only by aging but also by the intrinsic properties of the restorative material (Table 2).

### Influence of fatigue aging on color stability

Following fatigue aging , IPS e.max CAD ceramic veneers showed the lowest color change value (ΔΕ_00_) and thus the best color stability, while Cerasmart veneers showed the highest color change value, thus displaying the poorest color stability (Table 3). The specimens of glass ceramic groups had ΔΕ_00_ mean values lower than the values for specimens of hybrid ceramic groups. In addition, the color difference (ΔΕ_00_) values for glass ceramic groups were below the 3.3 acceptability threshold, meaning that both groups showed clinically acceptable color change after aging . In contrast, the hybrid ceramic groups had ΔΕ_00_ values higher than the 3.3 threshold, indicating clinically unacceptable color changes. The Post hoc Tukey test revealed statistically significant differences between the ΔΕ_00_ mean values for all groups except between the two glass ceramic groups EC and CT. A statistically significant difference was observed between group VE and group CS, with group CS exhibiting a statistically significant higher color change value (Table 3).

**Table 3:** Color differences (ΔE₀₀) for all tested groups after fatigue aging, with Post hoc Tukey multiple-group comparisons.

Group	ΔΕ_00_	
	Mean	SD
**EC**	2.34^A^	0.11
**CT**	2.70^A^	0.22
**VE**	3.45^B^	0.15
**CS**	4.79^C^	0.64

Group EC: IPS e.max CAD veneers; Group CT: Cerec Tessera veneers; Group VE: Vita Enamic veneers; Group CS: Cerasmart veneers. ^ABC^For significance of the Post hoc Tukey test: Mean values with the same superscripted letters represent a non-significant difference, and the different superscripted letters represent a significant one. SD: Standard Deviation. *Significance at *p*-value ≤ 0.05.

The applied fatigue aging protocol led to a significant decrease in values of L* (darker) and a significant increase in both values of a* (more reddish) and b* (more yellowish) for all tested specimens. Within-group comparisons between baseline (non-aged control) and post-aging measurements, using Paired *t*-test, demonstrated statistically significant differences for all color coordinates in all groups (*p* < 0.001), confirming that artificial aging significantly altered the color characteristics of all veneer–cement–substrate assemblies (Table 4).

**Table 4:** Comparison of individual color parameters measured before and after fatigue aging for all tested groups.

Group	Color parameter		Mean	SD	Paired *t*-test
**EC**	L*	**Pre**	68.52	0.86	*t* = -41.25 *p* < 0.001*
		**Post**	66.79	0.80	
	a*	**Pre**	0.45	0.39	*t* = 26.48 *p* < 0.001*
		**Post**	1.66	0.46	
	b*	**Pre**	7.20	0.82	*t* = 29.83 *p* < 0.001*
		**Post**	8.63	0.83	
**CT**	L*	**Pre**	57.91	0.29	*t* = 33.17 *p* < 0.001*
		**Post**	55.62	0.38	
	a*	**Pre**	0.50	0.16	*t* = 22.80 *p* < 0.001*
		**Post**	1.68	0.23	
	b*	**Pre**	7.71	0.71	*t* = 32.08 *p* < 0.001*
		**Post**	8.53	0.94	
**VE**	L*	**Pre**	72.31	0.75	*t* = -41.08 *p* < 0.001*
		**Post**	69.52	0.87	
	a*	**Pre**	0.46	0.67	*t* = 36.33 *p* < 0.001*
		**Post**	2.17	0.70	
	b*	**Pre**	6.08	1.31	*t* = 34.12 *p* < 0.001*
		**Post**	8.17	1.36	
**CS**	L*	**Pre**	72.08	1.07	*t* = -39.73 *p* < 0.001*
		**Post**	69.30	1.50	
	a*	**Pre**	0.45	0.44	*t* = 37.66 *p* < 0.001*
		**Post**	2.86	0.61	
	b*	**Pre**	2.39	0.65	*t* = 29.03 *p* < 0.001*
		**Post**	6.10	1.18	

Group EC: IPS e.max CAD veneers; Group CT: Cerec Tessera veneers; Group VE: Vita Enamic veneers; Group CS: Cerasmart veneers. Pre: before aging (baseline, control), Post: after aging . SD: Standard Deviation.

* Significance at *p*-value ≤ 0.05.

### Influence of fatigue aging on translucency

In comparison to other materials, IPS e.max CAD specimens showed the highest translucency behaviour both before (at baseline) and after the aging process (Table 5). Conversely, Vita Enamic specimens had the lowest translucency values before and after aging . Within-group comparisons between baseline (non-aged control) and post-aging measurements, using Paired *t*-test, demonstrated statistically significant decrease in TP values for all groups (*p* < 0.001).

**Table 5:** Comparison of translucency parameter measured before and after fatigue aging for all tested groups.

Group	TP	Mean	SD	Paired *t*-test
**EC**	**Pre**	26.82	0.42	*t* = 14.32 *p* < 0.001*
	**Post**	24.95	0.56	
**CT**	**Pre**	25.37	0.76	*t* = 8.45 *p* < 0.001*
	**Post**	23.64	0.87	
**VE**	**Pre**	25.02	0.90	*t* = 12.15 *p* < 0.001*
	**Post**	23.24	1.20	
**CS**	**Pre**	26.17	1.09	*t* = 13.02 *p* < 0.001*
	**Post**	23.77	1.10	

Group EC: IPS e.max CAD veneers; Group CT: Cerec Tessera veneers; Group VE: Vita Enamic veneers; Group CS: Cerasmart veneers. Pre: before aging (baseline, control), Post: after aging . SD: Standard Deviation; TP: translucency parameter.

* Significance at *p*-value ≤ 0.05.

## Discussion

The demand for optimal esthetics with minimal tooth preparation has increased the use of CAD/CAM-fabricated ceramic laminate veneers, particularly lithium disilicate glass ceramics and hybrid ceramic materials [26, 27]. The stability of optical properties, especially color and translucency, is critical for the long-term esthetic performance of these ceramic veneers. Therefore, artificial aging protocols simulating the oral environment are commonly used to evaluate these properties *in vitro* [28].

This study investigated the effect of AAA on the color stability and translucency of minimally invasive ceramic laminate veneers fabricated from contemporary glass ceramic and hybrid ceramic CAD/CAM materials. The null hypothesis, which assumed that artificial aging would not significantly influence the optical properties of the tested materials, was rejected. The applied protocol of artificial aging significantly affected both color stability and translucency of all tested materials. It resulted in darker, more reddish, and more yellowish color behaviour, accompanied by a reduction in translucency. Using the CIEDE2000 (ΔE_00_) color difference formula and considering the clinical acceptability threshold set at ΔE_00_ = 3.3, the color changes were clinically acceptable for glass ceramic materials but exceeded this threshold and thus clinically unacceptable for hybrid ceramic materials.

Standardisation of the underlying substrate is essential when evaluating thin ceramic veneers, as substrate shade can significantly influence the final optical outcome [28, 29]. Although natural teeth may provide a more clinically relevant background, they were not used in the present study as substrates due to the inherent variability in tooth shade, translucency, age-related changes, and structural variations, which could introduce uncontrolled variables and compromise experimental standardisation. Instead, a composite substrate with a standardised A2 dentin shade was used to minimise variability and improve the reproducibility of the *in vitro* measurements while simulating a commonly encountered dentin shade in clinical situations [26, 29]. However, it should be acknowledged that composite substrates cannot fully replicate the complex optical behaviour of natural teeth, particularly the layered structure of enamel and dentin and their light-scattering properties. Therefore, while composite substrates enhance methodological standardisation, natural teeth may provide greater biological relevance [29].

Objective color measurement using spectrophotometers provides more reliable and reproducible results than visual assessment [30, 31]. In this study, the Vita Easyshade V system was used, avoiding edge-loss error when the 5 mm plain recording tip was applied to the 8 mm flat, smooth specimens. Although this device is primarily designed as a clinical spectrophotometer, it was used under standardised laboratory conditions with repeated measurements to enhance reliability [20, 24]. Clinical spectrophotometers offer several practical advantages, including portability, ease of use, rapid measurement, and the ability to directly determine tooth shade using dental color coordinates and shade guide systems. In addition, their design allows for direct intraoral use, which facilitates translation of research findings to clinical practice [30]. Nevertheless, clinical spectrophotometers may present certain limitations, including sensitivity to probe positioning, specimen surface characteristics, and ambient light conditions. Despite these limitations, previous studies have demonstrated that the Vita Easyshade system provides reliable and reproducible color measurements when standardised measurement protocols and repeated readings are employed [20, 24, 32].

Laboratory spectrophotometers and colorimeters are commonly used alternatives for color evaluation in *in vitro* studies, each presenting specific advantages and limitations [33, 34]. Laboratory spectrophotometers generally provide high spectral resolution, precise wavelength analysis, and excellent measurement stability under controlled illumination conditions, which may enhance the accuracy of color measurements. However, these devices are often expensive, require more complex calibration procedures, and are less practical for routine dental shade determination [33]. Colorimeters are simpler and faster to operate and are less sensitive to minor positioning errors; however, they measure only tristimulus color values rather than the full spectral reflectance, which may limit their ability to detect subtle color differences [34].

Color differences were calculated using the CIEDE2000 (ΔE₀₀) formula, which offers improved perceptual uniformity and greater clinical relevance compared with the conventional CIELAB system [31, 35, 36]. There is a controversy in the literature regarding values of ΔE that are acceptable clinically. In this study, and commonly reported in the literature, ΔE values less than 1.0 were deemed invisible to the naked eye. Values of ΔE between 1.0 and 3.3 were deemed detectable by trained staff but still accepted clinically. Values of ΔE more than 3.3 were deemed perceptible to untrained individuals and hence regarded as unacceptable clinically [31, 35].

The applied thermomechanical aging protocol was designed to simulate approximately 1 year of clinical service [21–24]. In agreement with previous studies, aging significantly reduced L* values and increased a* and b* values across all materials [12]. These color changes may be attributed to alterations in both the ceramic materials and the resin cement components during aging . They cannot be attributed exclusively to the veneer materials, as the experimental design intentionally evaluated a clinically relevant veneer–cement–substrate complex. Consequently, the results represent the combined optical effects of the ceramic material, resin cement, and underlying composite substrate [11].

Regarding color stability after aging , glass ceramic materials exhibited significantly lower color changes compared with hybrid ceramic materials. IPS e.max CAD demonstrated the lowest ΔE₀₀ values, followed closely by Cerec Tessera, both remaining within clinically acceptable limits. These findings are consistent with previous studies reporting superior color stability of lithium disilicate-based ceramics after aging [35, 37–41]. In contrast, hybrid ceramic materials, particularly Cerasmart, showed significantly higher color changes exceeding the clinical acceptability threshold. This behaviour can be explained by their higher polymer content with greater susceptibility to water sorption, hydrolytic degradation, and resin matrix discoloration [38, 39, 42–44]. This may explain why Cerasmart specimens with a higher organic polymer component (29 wt%) exhibited a post-aging color difference value significantly greater compared to the other tested materials, including Vita Enamic specimens that have organic content about half of Cerasmart (14 wt%).

Observed color changes, particularly in thin ceramic veneers, may also be partially attributed to the resin cement used. Light-cured resin cements containing photosensitive components may undergo chemical degradation during aging , leading to increased yellowing over time. This effect is more pronounced in highly translucent restorations where the cement color can influence the overall appearance [11]. As stated by the respective manufacturer, VITIQUE esthetic resin cement is composed primarily of the poorly stain-resistant Bis-GMA (bisphenol Aglycidyl methacrylate) monomer, with chemical structure groups (hydroxyl and ester groups) that are prone to hydrolysis with water. As this cement ages, the water sorption characteristics of the resin monomers may contribute to differences in the degree of color stability. Hydrolytic degradation and hygroscopic effects (failure of the chemical bonds in the resin cement or the material’s softening due to water exposure) are considered determinant factors for color variation in resin materials [36, 45].

Translucency depends on several factors, including crystalline structure, chemical composition, grain size, and material thickness [46]. In this study, the elements that could affect the TP were controlled, as all specimens were prepared from CAD/CAM blocks of the same shade (HT, A2/2M2) and thickness (0.5 mm) [42]. IPS e.max CAD showed the highest translucency values, likely due to its glassy matrix and favourable refractive index, reducing light scattering and allowing light to penetrate the material and reflect the background color. Conversely, Vita Enamic exhibited the lowest translucency, possibly due to its higher alumina content and polymer-infiltrated structure [39, 46]. After aging , translucency significantly decreased for all materials, indicating increased opacity, consistent with previous findings [41, 42].

Despite efforts to simulate clinical conditions, this *in vitro* study has limitations. The study evaluated the combined optical behaviour of the veneer–cement–substrate complex; therefore, the individual contribution of each component to the observed changes in color and translucency could not be isolated. In addition, the experimental design did not allow identification of which specific stage of the accelerated thermomechanical aging protocol was responsible for these changes. Furthermore, the use of disc-shaped specimens, a single veneer thickness, one cement shade, and axial loading may not fully replicate the complexity of the oral environment. Surface gloss and roughness changes were also not quantitatively assessed. These factors should be considered when interpreting the results. Future studies should address these variables to further elucidate aging -related optical changes in ceramic laminate veneers, and long-term clinical trials should be conducted to further validate the present findings.

## Conclusions

Based on the results of this *in vitro* study, the following conclusions were drawn:

1. Accelerated thermomechanical aging significantly affected the color stability and translucency of all tested ceramic laminate veneer materials.
2. Glass ceramic materials (IPS e.max CAD and Cerec Tessera) demonstrated superior color stability compared with hybrid ceramic materials, with color changes remaining within clinically acceptable limits.
3. Hybrid ceramic materials, particularly Cerasmart, exhibited greater susceptibility to aging-related color changes and translucency reduction.
4. All tested materials showed a significant decrease in translucency following artificial aging .

## Data Availability

The datasets used and/or analysed during the current study are available from the corresponding author upon reasonable request.
